# Anatomical study for the treatment of proximal humeral fracture through the medial approach

**DOI:** 10.1186/s13018-021-02897-2

**Published:** 2022-01-17

**Authors:** Hao Xiang, Yan Wang, Yongliang Yang, Fanxiao Liu, Qingsen Lu, Lingpeng Kong, Mingzhen Li, Yong Han, Fu Wang

**Affiliations:** 1 Department of Orthopedics, Shandong Provincial Hospital, Cheeloo College of Medicine, Shandong University, No. 324, Jing Wu Road, Jinan, 250021 China; 2 Medical Laboratory Diagnosis Center, Jinan Central Hospital, 105 Jie Fang Road, Jinan, 250013 China; 3 Department of Orthopedics, Shandong Provincial Hospital Affiliated to Shandong First Medical University, 324 Jing Wu Road, Jinan, 250021 China

**Keywords:** Proximal humeral fractures, Medial approach, Anterior humeral circumflex artery

## Abstract

**Background:**

The treatment of complex 3- and 4-part proximal humeral fractures has been controversial due to numerous postoperative complications. With the further study of medial support and blood supply of humeral head, new techniques and conception are developing. The study aims to illustrate the medial approach of the proximal humeral fracture through cadaver autopsy.

**Method:**

Upper limbs from 19 cadavers have been dissected to expose the shoulder joint. We selected the coracoid process as the bony reference. Vernier caliper will be used to measure the following data, including distance from coracoid process to circumflex brachial artery, distance between anterior humeral circumflex artery (ACHA) and posterior circumflex brachial artery (PCHA) and their diameters. Assessment included the characteristics of the vascular supply around the humeral head, identification of the structures at risk, quality of exposure of the bony structures, and feasibility of fixation.

**Results:**

The medial approach is appropriate in 86.84% anatomical patterns. Between the lower part of the shoulder capsule and the insertion of conjoined tendon, the bony surface exposed was limited by the interval between ACHA and PCHA. An interval of 2 to 3 cm (24.29 ± 3.42 mm) was available for medial plate. ACHA (49.35 ± 8.13 mm, 35.14–68.53 mm) and PCHA (49.62 ± 7.82 mm, 37.67–66.76 mm) were about 5 cm away from the coracoid process. Risk structures including ACHA and PCHA originate in common, PCHA originated from the deep brachial artery (DBA), the presence of perforator vessels, musculocutaneous nerve intersects with ACHA, the diameter of PCHA: ACHA < 1.5. In 13.15% anatomical patterns, this risk structure should be taken seriously.

**Conclusion:**

The medial approach opens a new perspective in the optimal management of complex fractures of proximal humerus. Anatomical research proves that the medial approach is feasible. The interval between ACHA and PCHA is suitable for placement. Anatomical pattern and indication have been discussed, and we hypothesized that ACHA has been destroyed in complex PHFs. With further studies on the anatomy and mechanism of injury, the development of more clinical cases will be an important work of our institution in the future.

## Introduction

Proximal humeral fractures (PHFs) are the seventh most frequent fractures in adults, and the third most frequent in the upper limb, with the prevalence varies from 4 to 10% of all fracture types. In patients aged older than 40 years, a linear increase is present, and only less than wrist and femoral neck fractures in the elderly population (> 65 years) [1–3]. At present, the prevalence of high-energy trauma is decreasing while traumas on osteoporotic bone are increasing [4]. Complex displaced PHFs will occur more often in older women with comorbidities [5, 6]. With the arrival of the ageing of population in China, the rapid increase in PHFs is beyond doubt.

Surgical interventions for PHFs including open reduction and internal fixation (ORIF), intra-medullary nailing (IMN) and reverse shoulder arthroplasty (RSA). In a retrospective study of PHF, the locking plate fixation is the most common procedure (48.3%) of surgical procedures, followed by IMN (20.0%) and RSA (5.6%) [7]. Although the IMN had a lower complication rate compared to the locking plate group in the treatment of 2-part fractures in a prospective randomized study [8], the reoperation rate of 4-part fractures was significantly higher than 2- and 3-part because of numerous complications [9–11]. For shoulder arthroplasty, tuberosity nonunion remains a concern for hemiarthroplasty. RSA had significantly less postoperative external rotation versus ORIF and hemiarthroplasty [12]. So, the prognosis is still controversial due to the number of cases and prosthesis revision. The locking plates are still the mainstream in treating PHF because locking head screws inserted bi-directionally exhibit increased pull out strength in the metaphyseal bone of the humeral head [13]. If ORIF was adopted, the most important factor for favorable results in the treatment of complex 3-part or 4-part humerus fractures is anatomic reduction [14].

The treatment for complex PHFs in older patients is still controversial because of osteoporosis and complications [15–17]. And the most common complications included varus malunion (16.3%), AVN (10.8%), screw perforation into the joint (7.5%) [18]. Loss medial cortical buttress from fracture comminution is the most common cause for varus malunion. Recent studies have found the stability has significant correlation with the medial column [19, 20]. As the proximal humerus is an eccentrically loaded joint, the alignment relies almost entirely on the plate if the anatomical reduction is not achieved. In mechanical experiment, the medial cortex contact shows better result in fatigue life than screw fixation group [21]. To reinforce the medial column, surgeon came up with several methods: Use calcar screws or endosteal implant; Impact the shaft into the humeral head; Insert an intramedullary fibular strut graft; Use dual plate fixation. The medial plate provided a firm buttress. Medial buttress plating through deltopectoral approach for PHFs has been reported [22]. But the affection of the blood supply to the head, especially the AHCA, remains controversial. And the selection of size and type of medial plate also needs further study. Good prognosis has been obtained in both biomechanical evaluation and clinical practice in our institution [23, 24]. However, some experts believe that the medial approach will sacrifice the ACHA. Therefore, this paper will focus on the anatomy and precautions of the medial approach.

## Materials and methods

The protocol of this study was approved by the Committee on Medical Ethics of Shandong Province Hospital affiliated to Shandong University. 19 frozen cadaveric paired upper limbs from voluntary donor specimens were used, which included both 12 males and 7 females. All cadavers were fixed in 8% formalin and preserved in 30% ethanol. When the dissection process begins, the specimens were preserved at a low temperature of about 2 °C. In each body, both left and right shoulders were carefully dissected. Mean donor age was 68.8 years (range 61–87 years) and mean donor height was 168.8 cm (range 153–183 cm). All limbs were examined for the absence of signs of previous surgery, trauma, or obvious gross deformity.

**Fig. 1:**
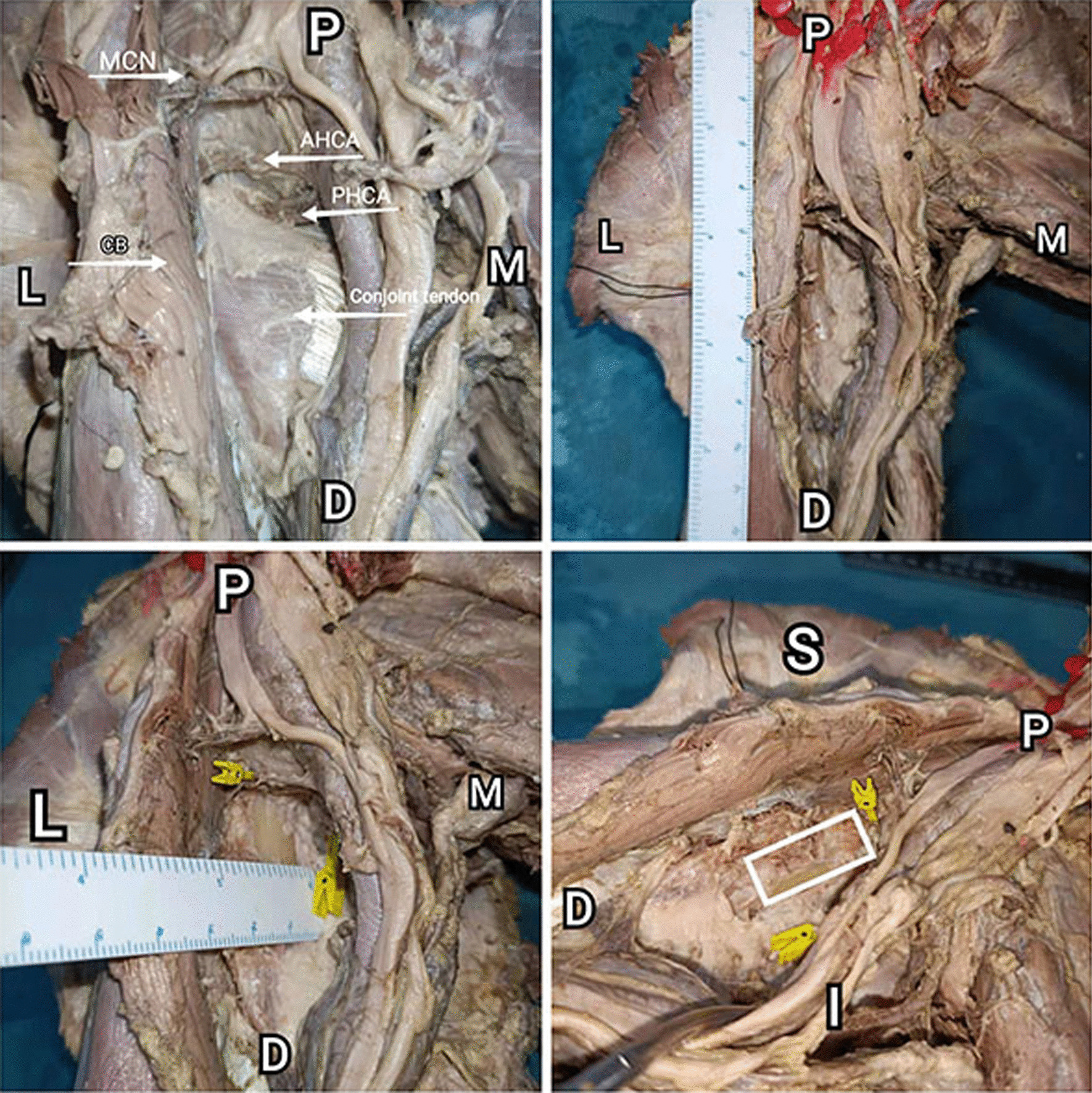
Front view of shoulders (Right) (P: proximal D: distal M: medial L: lateral). ACHA: Anterior circumflex humeral artery; PCHA: Posterior circumflex humeral artery; MCN: Musculocutaneous nerve; CB: Coracobrachialis. The conjoint tendon lies between the coracobrachialis and the brachial plexus. (The pectoralis major was removed for a better view). The distance from the coracoid process to the ACHA was measured. After conjoint tendon removed, the bone surface of proximal humerus is exposed.

The upper limbs were placed with the arm abducted to 60° on average in supine position. All specimens have been dissected through a medial incision to expose the shoulder joint [24]. The incision began proximally from the front end of the armpit and then extended toward the medial epicondyle of the humerus with a length of approximately 12 cm. The insertion of the pectoralis major tendon onto the humerus was transected and reflected for better visualization, which can be pulled upward and medially during the operation instead of transection. After dissecting and remove of superficial fascia tissue, the short head of biceps brachii and coracobrachialis muscles were exposed and pulled laterally with the musculocutaneous nerves. The brachial blood vessels and the rest of brachial plexus was identified and pulled medially. Between the conjoined tendon of the latissimus dorsi and teres major muscles and the lower border of the shoulder capsule, the medial side of the proximal humerus can be well exposed after the conjoint tendon is dissected (Fig. 1).

**Fig. 2:**
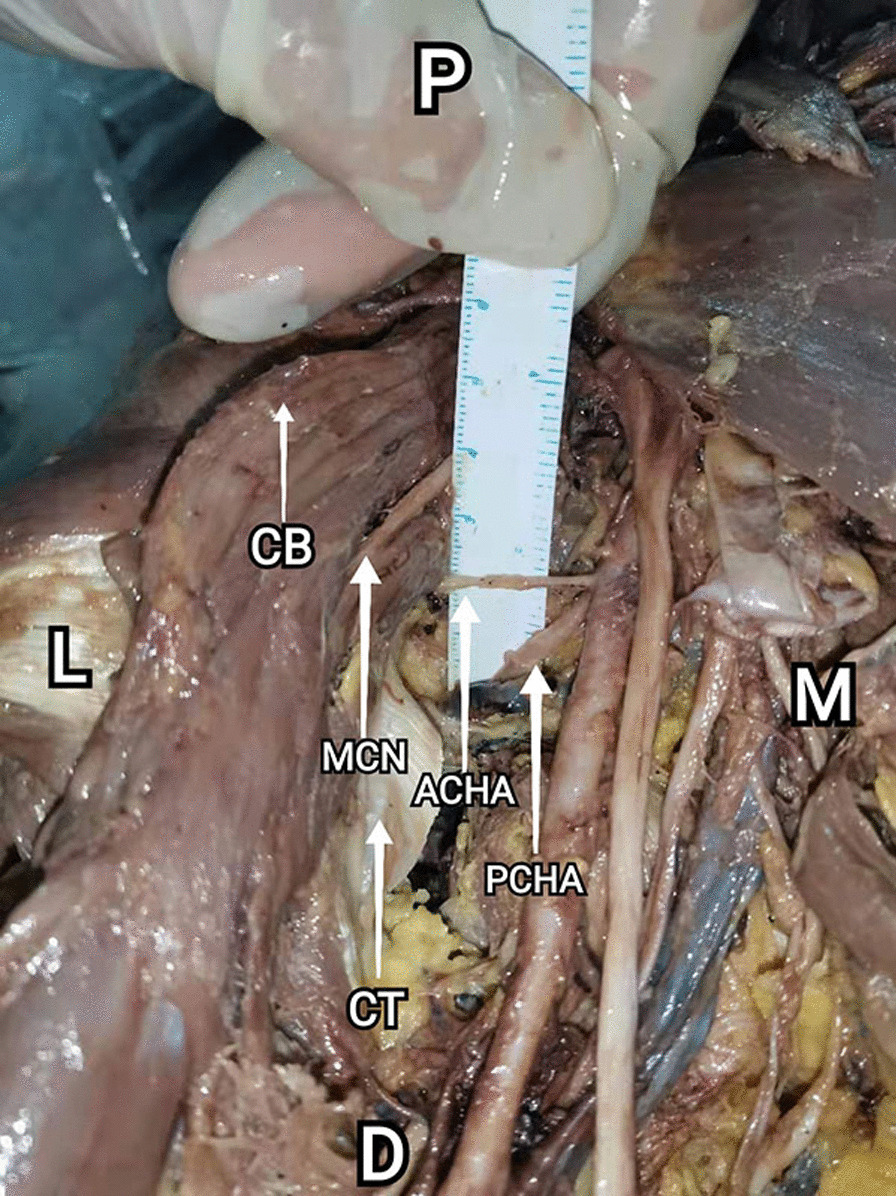
Inferior aspect of axilla (Right) (P: proximal D: distal M: medial L: lateral). CT: Conjoint tendon of the latissimus dorsi and teres major muscles; ACHA: Anterior circumflex humeral artery; PCHA: Posterior circumflex humeral artery; MCN: Musculocutaneous nerve; CB: Coracobrachialis. Structures at risk: ACHA, PCHA, musculocutaneous nerve. The interval between ACHA and PCHA is the area that suitable for the placement of the medial plate

Three structures require special attention due to their relative transverse configuration, including ACHA, PCHA and musculocutaneous nerve. The ACHA, PCHA and musculocutaneous nerve was identified, dissected, and their paths were traced after bifurcating from the axillary artery and brachial plexus. The musculocutaneous nerve was seen to travel laterally and anteriorly and pass through the coracobrachialis irregularly. The ACHA was seen to travel laterally under the tendon of the long head of the biceps and terminates with smaller branches in the greater tuberosity. The PCHA was seen to travel laterally and posteriorly, travels with the axillary nerve, remaining superior to the latissimus dorsi tendon. The interval between ACHA and PCHA is the area that suitable for the placement of the medial plate (Fig. 2). Characteristics of the nerve and vascular risk have been described in result.

**Fig. 3:**
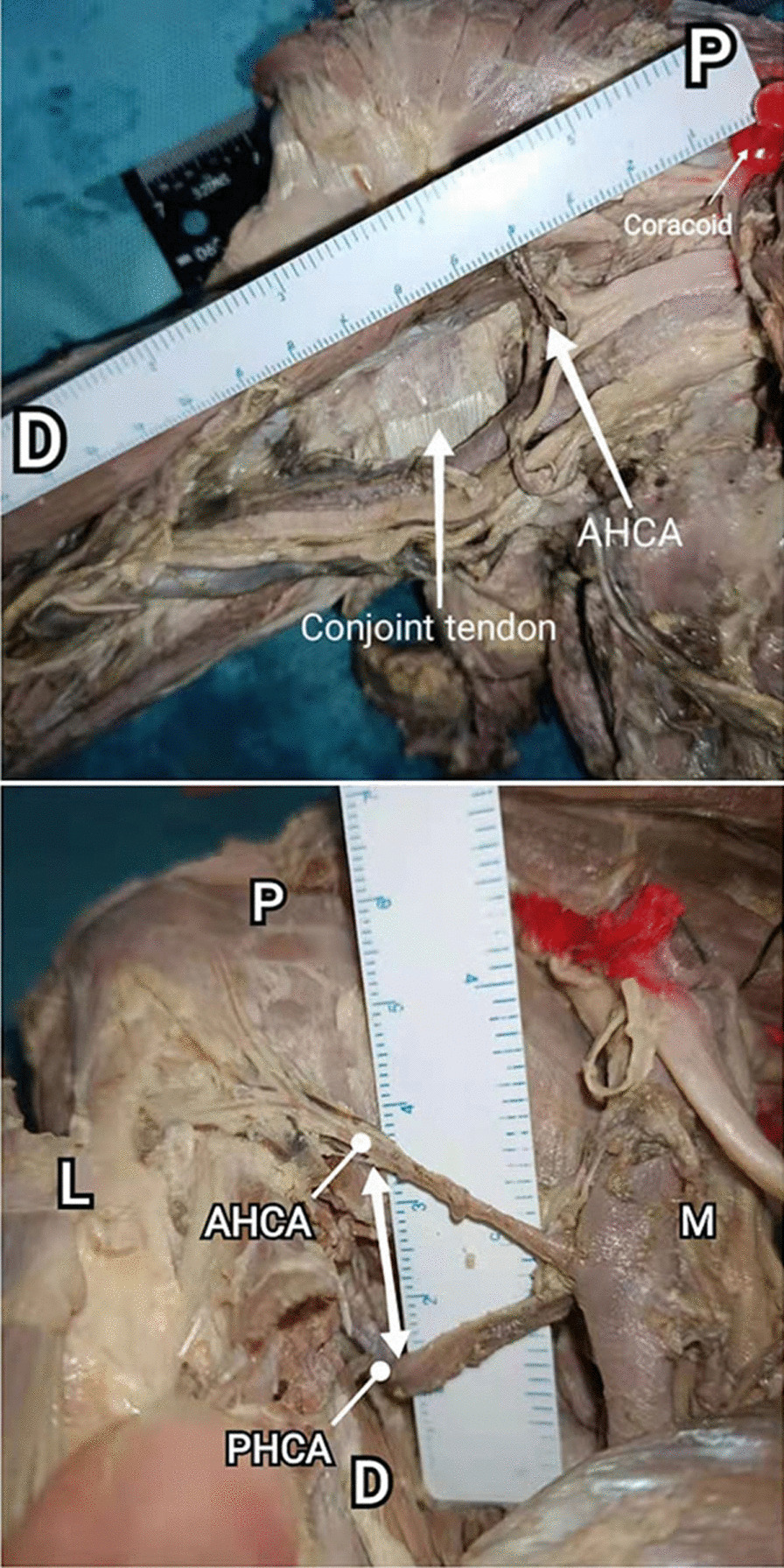
Measurement of data (Right) (P: proximal D: distal M: medial L: lateral). Distance from coracoid process to the ACHA and PCHA. Distance between ACHA and PCHA. Diameter of ACHA and PCHA. The interval between ACHA and PCHA was measured

The coracoid process was tagged with pin as the landmark. All anatomic relationships were measured using ruler placed in situ and marked to measure the length of each respective distance. All measurements were confirmed by a minimum of two observers. Vernier caliper has been used to measure the following data, including distance from coracoid process to the ACHA and PCHA, distance between ACHA and PCHA and the diameter of ACHA and PCHA after bifurcating from the axillary artery. The extent of bone surface available to place a plate is mainly limited by the distance between ACHA and PCHA. Thus, we measured the distance between ACHA and PCHA instead of the exposed bone surface. 14 limbs were used to expose the vessels distinctly and to measure the interval between ACHA and PCHA (Fig. 3).

Descriptive statistics were calculated including mean, standard deviations and range including minimum and maximum values. The data were further analyzed using a student’s paired t-test for analysis of the diameter of ACHA and PCHA, distance from coracoid to ACHA and PCHA, with statistical significance set at *p* < 0.05.

## Results

**Fig. 4:**
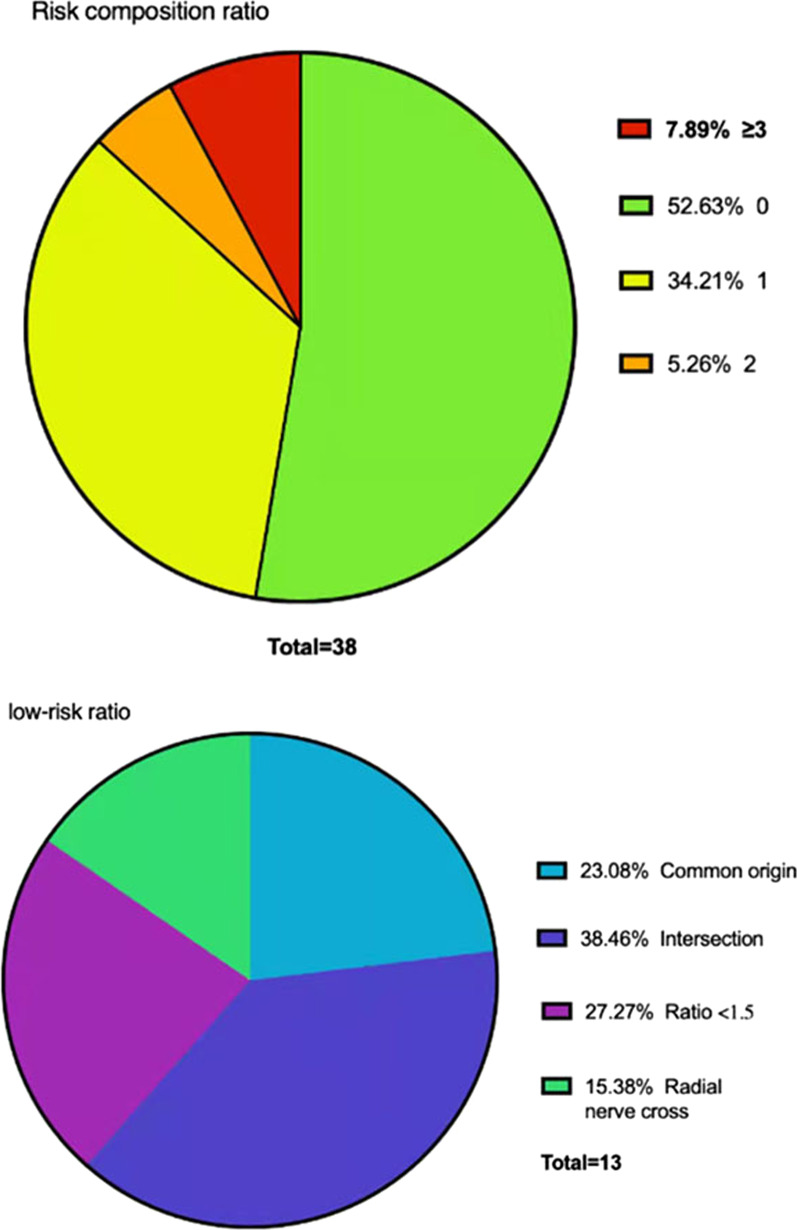
Proportion of risk score. 0 (52.63%), 1 score (34.21%): b (38.46%); c (27.27%); a (23.08%); d (15.38%). 2 (5.26%): b + c (2.63%); a + c (2.63%), ≥ 3 (7.89%): B + a (5.26%); A + b

Based on the following anatomical characteristics, we identified 4 relatively low risk structures and 2 relatively high-risk structures for the medial approach. Low risk structures accounted for 1 point, and high-risk structures accounted for 3 points. If the total score is less than 2, the placement of medial approach is practicable. Because of the anatomical characteristics of ACHA and some types of musculocutaneous nerves, there is a risk of injury. So we define low-risk structures, including ACHA and PCHA originate in common, the diameter of PCHA: ACHA < 1.5, musculocutaneous nerve intersects with ACHA, radial nerve cross between ACHA and PCHA. Due to variation of artery, arteries are susceptible to damage and intraoperative hemorrhage may be caused. Tourniquet cannot be used for hemostasis at the proximal end of humerus, which will lead to hemostasis and placement of fixation difficulty. So, we define relative high-risk structures, including PCHA originated from the deep brachial artery (DBA), the presence of perforator vessels. The proportion of 0, 1, 2 and 3 or above was 52.63%, 34.21%, 5.26% and 7.89%, respectively (Table 1; Fig. 4). In the low-risk group (1 score), musculocutaneous nerve intersecting with ACHA had the highest proportion of risk structures (38.46%), while the proportion of radial nerve cross between ACHA and PCHA was the lowest (15.38%).

**Table 1:** Risk of the medial approach

Score	Study subjects (*n* = 38) *N* (%)
0	20 (52.63)
1	13 (34.21)
2	2 (5.26)
≥ 3	3 (7.89)
*1 point:*	
a) AHCA and PHCA originate in common	
b) Musculocutaneous nerve intersects with ACHA	
c) Ratio of PCHA: ACHA < 1.5	
d) Radial nerve cross between ACHA and PCHA	
*3. points:*	
A. PCHA originated from the DBA	
B. Presence of perforator vessels

**Fig. 5:**
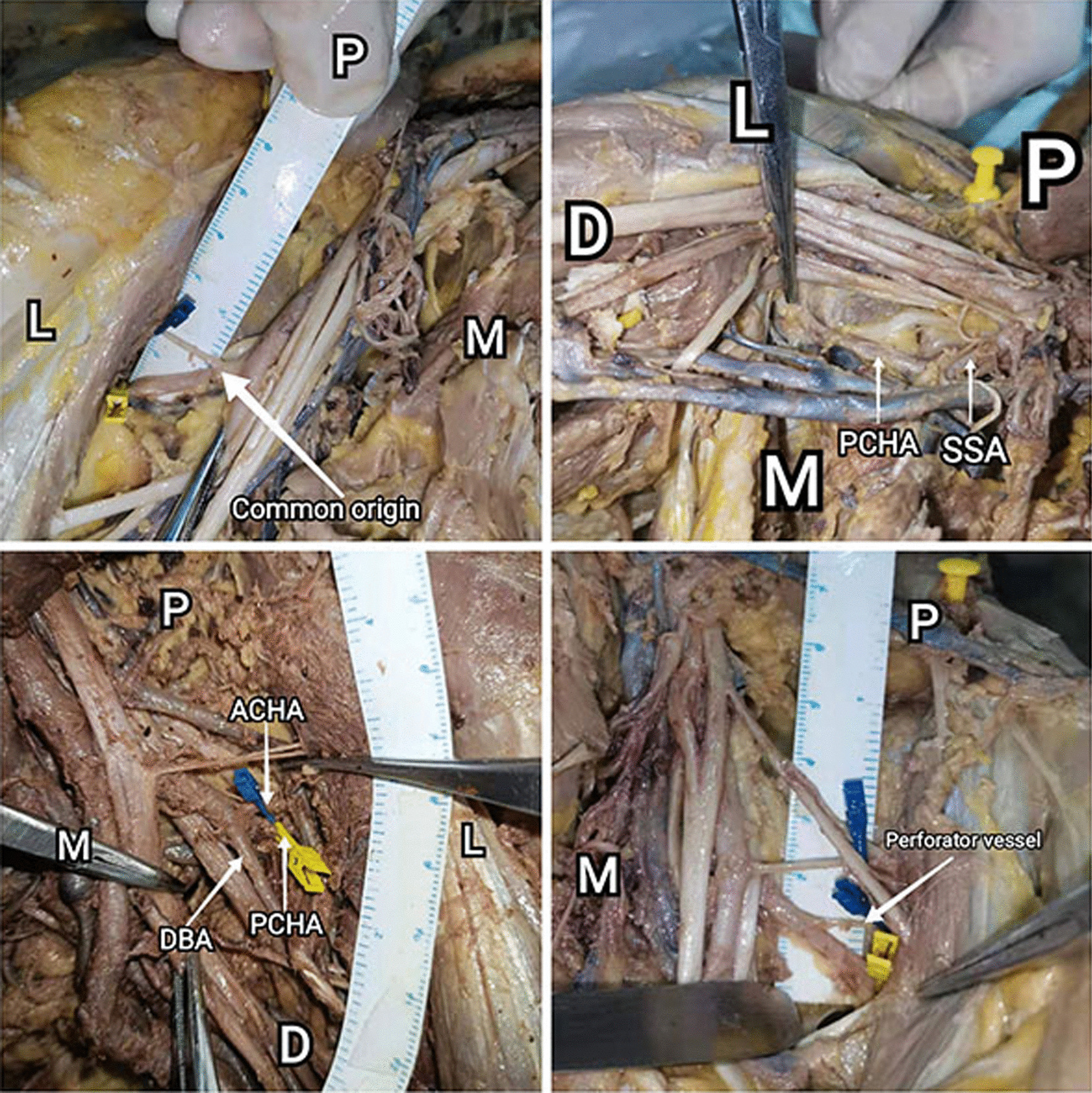
The variation of artery. **a** ACHA originated from the PCHA (common origin) (right), **b** ACHA originated from the subscapular artery (SSA) (right), **c** PCHA originated from the deep brachial artery (DBA) (left), **d** Branching arterioles in the direction of the coracobrachialis (left)

Normally, the ACHA originates from the anterolateral side of the axillary artery and passes through the coracobrachialis, with low mobility. When ACHA and PCHA originate in common, the interval between them is relatively fixed and the extent of exposure obtained by traction is limited (Fig. 5a). Generally, 84.3% ACHA and PCHA do not originate in common while 15.7% of cases originate in common, which led to the risk of injury to the ACHA when the PCHA was pulled to expose the operation area (Table 2). The detection and protection of ACHA is particularly critical in the medial approach.

**Table 2:** Characteristics of the vascular supply

		Study subjects (Total 38), *n* (%)
1. ACHA and PCHA have same origin	No	32(84.3)
	Yes*	6(15.7)
2. PCHA variation	Classical	33(86.8)
	SSA	4(10.5)
	DBA*	1(2.6)
3. Perforator vessel exist	No	36(94.7)
	Yes*	2(5.3)
4. PCHA: ACHA	Ratio ≥ 1.5	32(84.3)
	Ratio < 1.5*	6(15.7)

*: Relatively high-risk structure; *: Relatively low-risk structure

Classical: PCHA originated from the axillary artery

SSA: PCHA originated from the subscapular artery

DBA: PCHA originated from the deep brachial artery

**Fig. 6:**
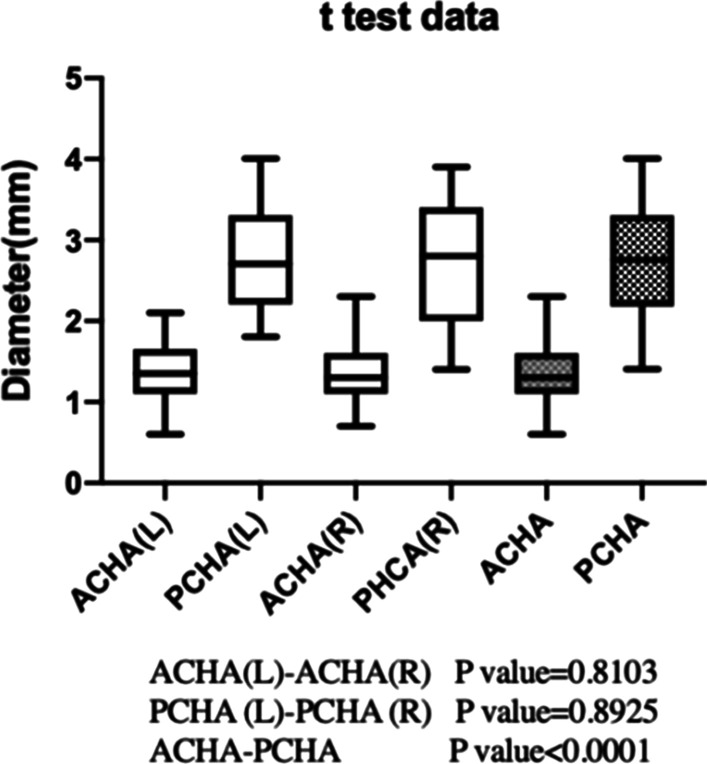
The diameter of the ACHA and PCHA. The mean diameters of the ACHA were 1.38 ± 0.39 mm (0.6 ~ 2.30 mm). The mean diameters of the PCHA were 2.74 ± 0.72 mm (1.4 ~ 4.00 mm). The mean ratio of the PCHA to ACHA is about 2.03 ± 0.68 (1.10 ~ 4.28)

A thicker ACHA may play a more important role in preventing avascular necrosis in PHFs. The mean ratio of the PCHA to ACHA is about 2.03 ± 0.68 (1.10 ~ 4.28). The mean diameters of the ACHA and PCHA were 1.38 mm (0.60–2.30 mm, SD 0.39 mm) and 2.74 mm (1.40–4.00 mm, SD 0.72 mm), respectively. The larger the ratio of the PCHA to ACHA, the less effective the ACHA will be. 1.5 is chosen as the standard. There are 6(15.8%) cases with a ratio less than 1.5 and 31 (81.6%) cases with a ratio greater than 1.5, among which 2 (4 cases) specimens had ratios less than 1.5 on both sides. For the diameter of ACHA and PCHA, there was no statistical difference between the left and right sides (Table 3, Fig. 6).

**Table 3:** Diameter of the arteries (*n* = 38)

		Minimum	Maximum	Mean	Std. deviation	*P* value
1	ACHA(L)	0.6	2.1	1.39	0.38	0.8103
	ACHA(R)	0.7	2.3	1.36	0.40	
2	PCHA(L)	1.8	4.0	2.75	0.64	0.8925
	PCHA(R)	1.4	3.9	2.72	0.78	
3	ACHA(T)	0.6	2.3	1.38	0.39	< 0.0001
	PCHA(T)	1.4	4.0	2.735	0.72	
4	PCHA: ACHA (in pair)	1.10	4.28	2.03	0.68	/

L(Left); R(Right); T(Total)

1, 2,3: Diameter of ACHA and PCHA (mm)

4: Diameter of PCHA: ACHA (in pair)

Variations in PCHA are common during measurement. Typically, PCHA originated from the axillary artery, as is classically described in 86.8% of cases in our research. Besides, PCHA originated from the subscapular artery (SSA) (Fig. 5b) in 10.5% of cases and originated from the deep brachial artery (DBA) (Fig. 5c) in 2.6% of cases. When PCHA originated from the subscapular artery (SSA), this variation results in a deeper and higher origin and course of PCHA. This variant is considered as the safer type. On the contrary, the variation that PCHA originated from the DBA may reduce the placement space of the medial plate.

No perforator vessels were found in 94.7% of the cases. A bare spot on the medial proximal humerus existed in the region between ACHA and PCHA. However, in 2 cases (5.3%) the PCHA gave off a branching artery in the direction of the coracobrachialis before penetrating the quadrilateral foramen (Fig. 5d). In the absence of perforators, the PCHA has a very high range of mobility, making it ideal for placement and operation of internal fixation. Perforator vessels to the coracobrachialis can be ligated during surgery; this requires a surgeon to be anatomically competent.

**Fig. 7:**
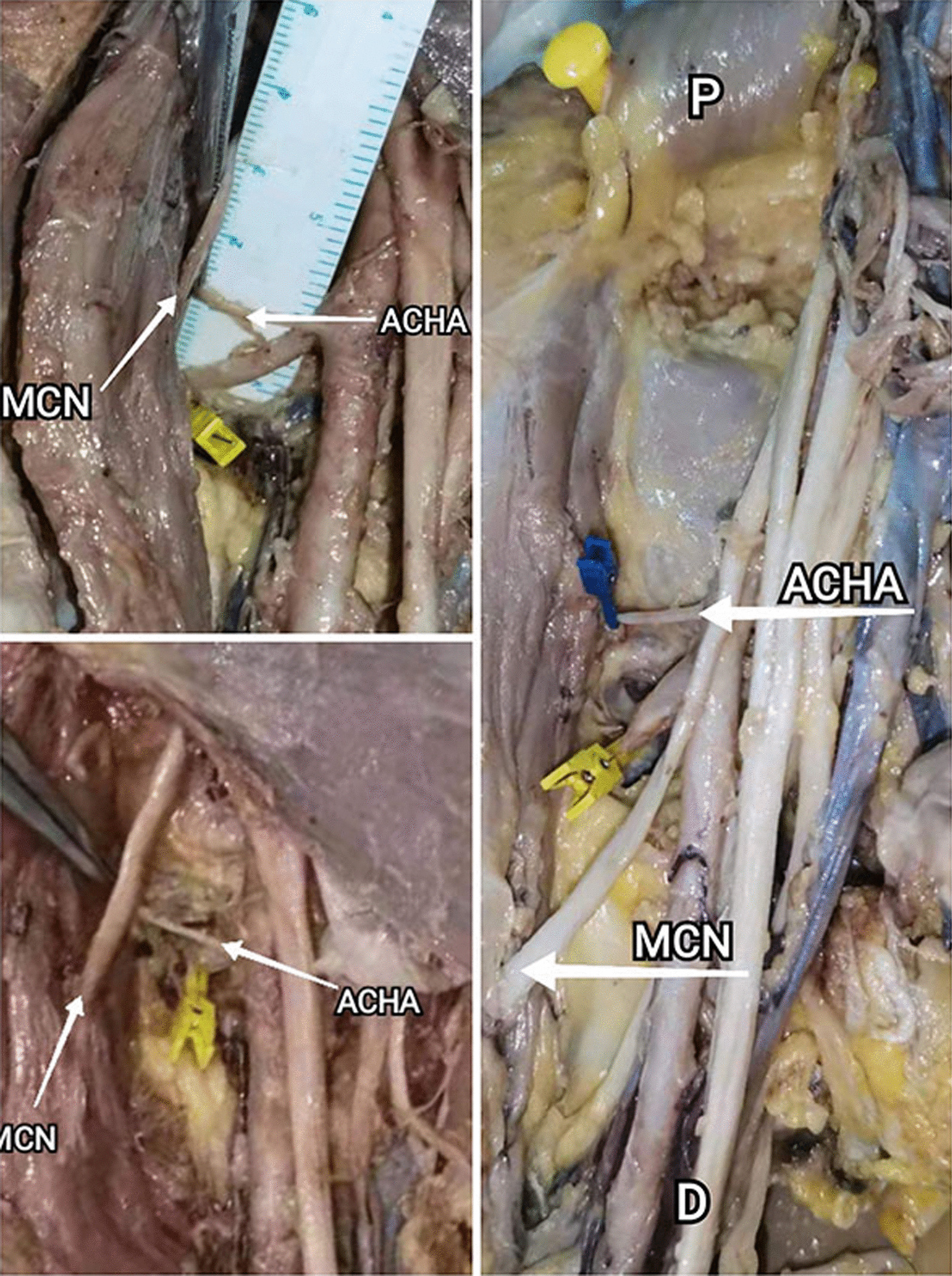
Relation between musculocutaneous nerve and circumflex brachial artery (Right). I: The point of afferent to the coracobrachialis is located proximal to the ACHA. II: The musculocutaneous nerve intersects with ACHA. III: The point of afferent to the coracobrachialis is located far distal to the ACHA

**Fig. 8:**
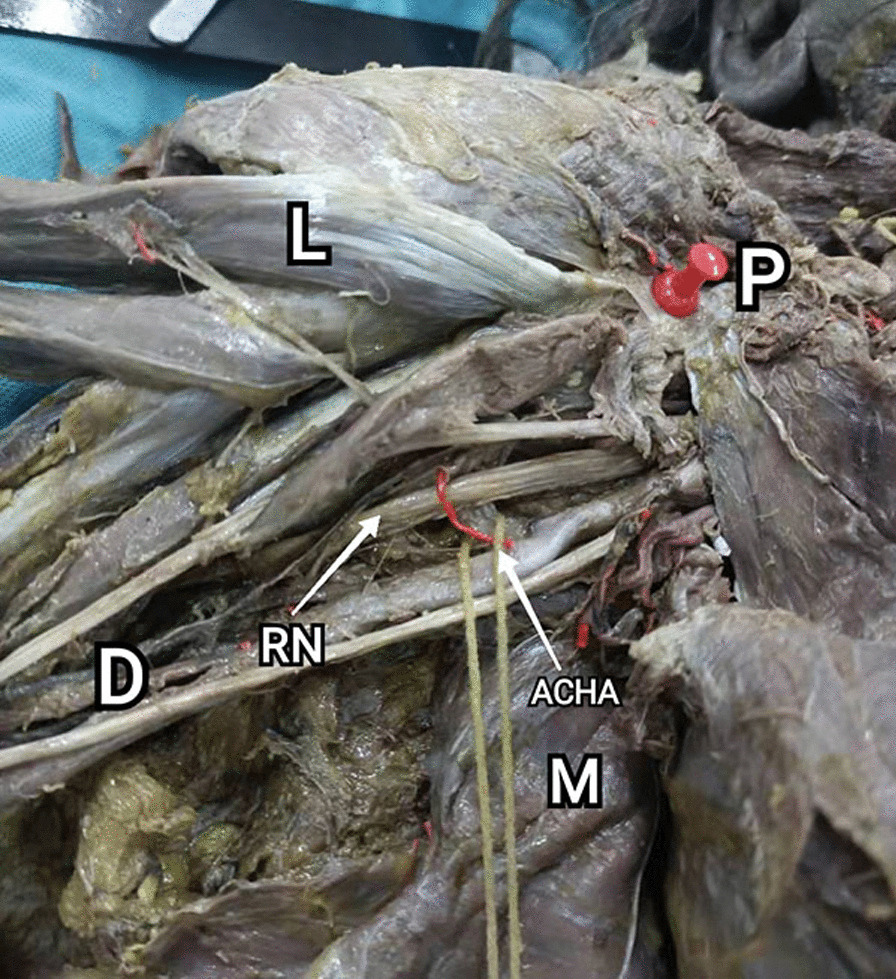
Radial nerve cross between ACHA and PCHA (Right)

Another risk structure is the musculocutaneous nerve intersects with ACHA. According to the anatomical relationship between musculocutaneous nerve and ACHA, we divide the musculocutaneous nerve into 3 categories (Table 4; Fig. 7). I (76.3%): The afferent point to the coracobrachialis is located proximal to the ACHA. II (18.4%): The musculocutaneous nerve intersects with ACHA. III (5.3%): The afferent point is located distal to the ACHA. Because the mucocutaneous nerve needs to be pulled laterally with the coracobrachialis, careful attention should be paid in type II. In contrast, type III has little effect on surgical area exposure because the musculocutaneous nerves tend to be extremely relaxed. In addition, the radial nerve was found to cross between ACHA and PCHA in 2 cases, which may reduce operating space (Fig. 8).

**Table 4:** Characteristics of the nerve

	Study subjects (Total 38), *n* (%)
*1. Musculocutaneous nerve*	
I	29(76.3)
II*	7(18.4)
III	2(5.3)
*2. Radial nerve cross between AHCA and PHCA*	
No	36(94.7)
Yes*	2(5.3)

*: Relatively low-risk structure

I: The afferent point to the coracobrachialis is located proximal to the ACHA

II: The musculocutaneous nerve intersects with ACHA

III: The afferent point is located distal to the ACHA

**Fig. 9:**
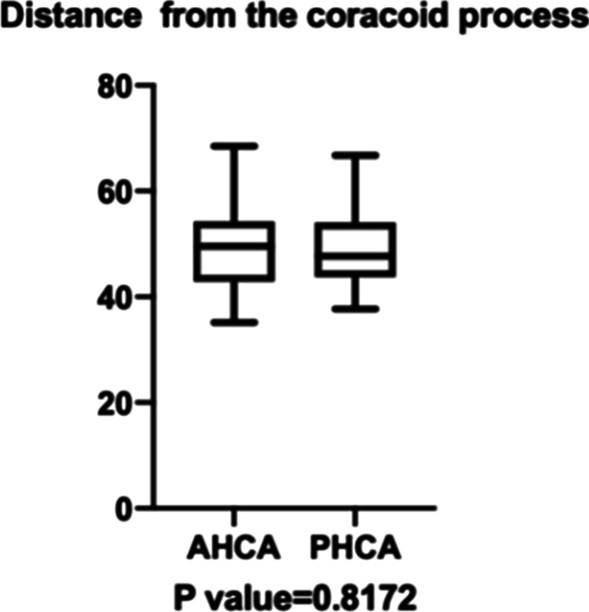
Distance from artery to bone marker (coracoid process) (38 upper limbs). The distance from the coracoid process to ACHA is 49.35 ± 8.13 mm (35.14–68.53 mm). The distance from the coracoid process to PCHA is 49.62 ± 7.82 mm (37.67–66.76 mm)

**Fig. 10:**
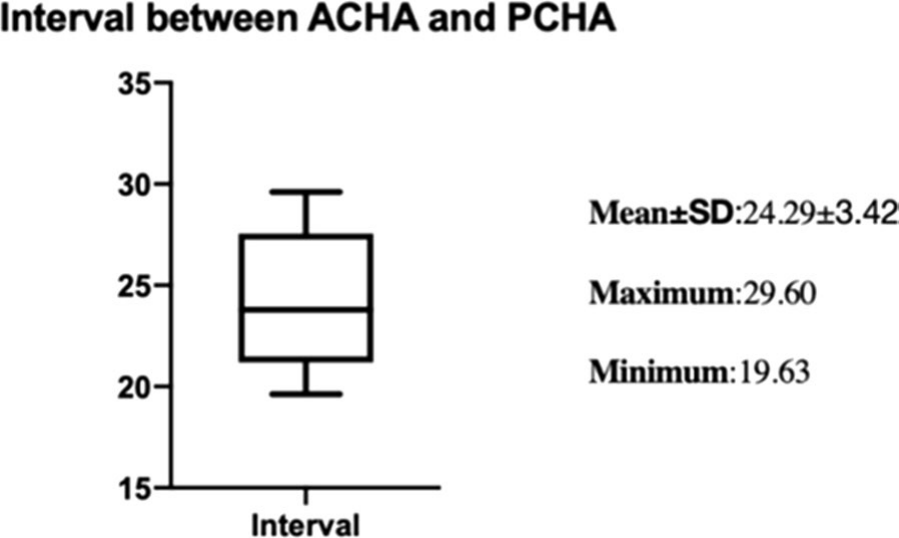
The average distance in 12 upper limbs between ACHA and PCHA was 24.29 ± 3.42 mm (19.63–29.60 mm). Two examples were abandoned.1) ACHA rupture; 2) ACHA and PCHA originate from DBA

The distance data are as follows. The distance from the coracoid process to ACHA is 49.35 ± 8.13 mm (35.14–68.53 mm). The distance from the coracoid process to PCHA is 49.62 ± 7.82 mm (37.67–66.76 mm). There was no statistical difference between ACHA and PCHA (*P* value = 0.8172) (Fig. 9). In 12 upper limbs, the interval between ACHA and PCHA was measured; the average distance was 24.29 ± 3.42 mm (19.63–29.60 mm) (Table 5, Fig. 10). Among the specimens measured, one PCHA originated from DBA. The distance between PCHA and ACHA is only about 5 cm, so it was not included in the data statistics. In addition, the ACHA of one specimen was cut off during measurement, and the data were invalid.

**Table 5:** Distance of the vascular supply (DISTANCE I and II, *n* = 38; DISTANCE III, *n* = 14)

	Maximum	Minimum	Mean	Std. deviation	*P* value
*DISTANCE I (mm)*					
Valid, 36(94.7)	68.53	35.14	49.35	8.13	0.8172
*DISTANCE II (mm)*					
Valid, 36(94.7)	66.76	37.67	49.62	7.82	
DISTANCE III (mm)	*n* = 14				/
Valid, 12(85.7)	29.60	19.63	24.29	3.42	

DISTANCE I: ACHA to coracoid process

Two examples were removed from the sample due to AHCA rupture

DISTANCE II: PCHA to coracoid process

Two examples were abandoned because PHCA originated prematurely from SSA

DISTANCE III: Interval between ACHA and PCHA

Two examples were abandoned. (1) ACHA rupture; (2) ACHA and PCHA originate from DBA

## Discussion

In this study, we describe a surgical approach allowing to address complex proximal humeral fractures involving medial side and summarize the anatomical characteristics of the medial approach. We found that through the medial approach, ACHA, PCHA and musculocutaneous nerve have a higher risk of injury due to their anatomical characteristics.

Through the medial approach, the medial side of the proximal humerus can be well exposed between the lower part of the shoulder capsule and the insertion of conjoined tendon. The approach permits direct vision reduction and medial support. But the extent of exposure is limited is limited by the interval between ACHA and PCHA. Our anatomical studies show that the average distance between ACHA and PCHA was 24.29 ± 3.42 mm (19.63–29.60 mm), which is sufficient to place the medial plate. Previous study shows the mean distances from the origin of PCHA and ACHA to the infraglenoid tubercle were 27.7 mm and 26.9 mm, respectively [25]. These data are instructive for surgeons to choose the size of medial plate.

**Fig. 11:**
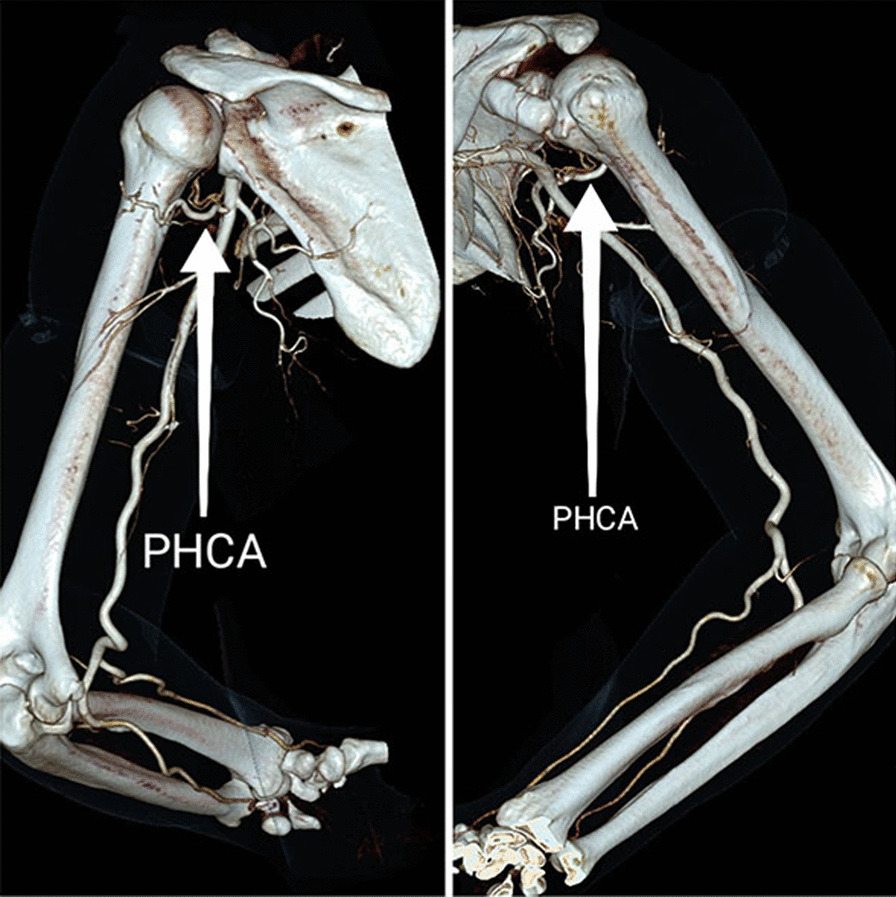
CTA of upper left limb. The ACHA is difficult to visualize in CTA

Once the medial approach is adopted, it is recommended to locate the ACHA and PCHA before operation. Method of guiding the quick access to ACHA by landmarks has been proposed [25]. In this study, the distance from ACHA and PCHA to coracoid was 49.2 mm and 50.2 mm. The data in our study are 49.35 ± 8.13 mm and 49.62 ± 7.82 mm, respectively, which is consistent with previous study. This technique provides favorable guidance for preoperative localization of ACHA. CT﻿A can be used to determine the continuity of artery before surgery but is often not used routinely due to its high cost and unclear development (Fig. 11). In addition, location of the ACHA by intraoperative ultrasound is possible due to the loose subcutaneous tissue in the medial upper arm as using intraoperative ultrasonography in treatment of acute achilleas tendon rupture yield less surgical time [26].

Based on the above observation, the interval between ACHA and PCHA is practicable for the placement of medial plate in 86.84% anatomical patterns. But the variation of the PCHA is also noteworthy. According to literature reports, the typical PCHA accounted for 77.1%, PCHA arises from SSA accounted for 12%, PCHA arises from DBA accounted for 8.4% [27]. These data in our observations are 86.8%, 10.5% and 2.6%, respectively. When the PCHA arises from the subscapular artery, its origin is located proximal to the typical type. We think it is safer because the deeper course of PCHA. But when it comes from the deep brachial artery, the distance between ACHA and PCHA is much shorter than the typical type (Fig. 5c). Besides, in 2 cases (5.3%) the PCHA gave off a branching artery before penetrating the quadrilateral foramen. These two relatively high-risk structures can lead to difficulties in intraoperative hemostasis and fixation placement. Constant vigilance is necessary during operation.

In our study, the diameter of PCHA was observed to be much larger than that of ACHA, which indicated that PCHA may play a more important role than ACHA in preventing AVN. The small diameter of the ACHA (0.3–2 mm) in comparison with that of the PCHA (1.2–5.5 mm) is also funded by other studies [24, 28, 29]. Earlier anatomic dissection studies indicated the vascularization of humeral head was mainly through the ACHA while the PCHA vascularized only a small part of the head [30]. But this result could not explain the absence of necrosis in the cases of severe fracture as the ACHA is vulnerable in such cases. PCHA seems to play a decisive role in blood supply to the humeral head in most of the recent studies. [28] [31]. Last, Natalie Keough et al. emphasized the variations exist for the course of the ACHA, which suggest a more significant contribution from the PCHA to the epiphysis [32]. In our study, a separate origin for the ACHA and PCHA was 84.3%. This is consistent with their study (ACHA = 76%; PCHA = 60%). So, we agree that PCHA provides dominant blood supply for the humeral head. Therefore, there is no need for excessive exposure of the PCHA during the operation.

Given the anatomical features of ACHA, we hypothesized that the integrity of ACHA has been lost in complex PHFs as the ACHA was firmly attached to the subscapularis tendon [33]. In previous study, no intact ACHA was found in all cases except 1 patient through deltopectoral approach [22]. The entry point of arcuate artery, which regard as an important intraosseous anastomosis, is in the outer upper quadrant of the humeral head [34]. This makes the ACHA vulnerable to damage in the deltopectoral approach. While the ACHA has been lost, extent dissection of surrounding soft tissues on the anterior side of the humerus may increase the risk of necrosis. That’s may explain why avascular necrosis of the greater tuberosity occurred in 2 patients through deltopectoral approach. Instead, the medial approach can detect and protect ACHA from its origination and does not require excessive dissection because of its loose subcutaneous tissue. In addition, with a medial approach, the plate can be covered by the conjoined tendon of the latissimus dorsi and teres major muscles rather than placed over it. Besides, the longitudinal incisions on the medial side contribute to the concealment of the incisions and have little effect on cutaneous blood supply and cutaneous nerves without any flap.

Compared to other nerves, the musculocutaneous nerve is the only nerve that requires special attention in medial approach. It is reported that shoulder abduction could protect the axillary nerve and radial nerve when working near the latissimus dorsi tendon insertion [35]. We found that the axillary nerve was generally located behind the PCHA, so the medial approach did not increase the risk of axillary nerve injury. The difficulty of internal fixation placement is influenced by its position with the ACHA. Based on the distance between its origin from the brachial plexus and its afferent coracobrachialis muscle. We divide the musculocutaneous nerves into three categories. Type I. The entry point is proximal to the ACHA. Type II. The entry point is located adjacent to the artery (musculocutaneous nerve intersects with ACHA). Type III. The entry point is located distal to the ACHA. In this research, 76.3% fits type I. As all the musculocutaneous nerve should be pulled laterally to facilitate the placement of the implant during surgery, so the type I is beneficial to surgery. In a study on the relationship of the musculocutaneous nerve, approximately 83% entry points that musculocutaneous nerve penetrates the coracobrachialis were shorter than 5 cm from the humeral head [36]. This is consistent with this article, and the proportion is even higher. Type III is also considered safe because of its relaxed tension and ease of retracting. When musculocutaneous nerve intersects with ACHA, attention should be paid when placing the fixation.

The conjoined tendon of the latissimus dorsi and teres major muscles needs to be cut off and sutured to cover fixation. But its function is almost unaffected. Modified L’Episcope procedure have been proposed. The follow-up shows that active internal rotation remained unchanged (7.6 ± 2.0 compared to 7.5 ± 2.4) [37]. So, the dissection of the conjoined tendon will not affect the function of the shoulder.

About indication, unstable medial cortical reconstruction have been proposed [22]. Beside 3- and 4-parts fractures, any medial cortical deficiency can be restored through a medial approach. In the case of coracoid process injury, dislocation and other injuries, related tissue repair can also be carried out through the medial approach under direct vision. Short calcar segment (8 mm), Disrupted medial hinge (2 mm dislocation), and some fracture pattern predict of ischemia of humeral head [38]. From our perspective, the imaging evidence is consistent with injury to the ACHA. In such cases, the medial approach is no longer limited by ACHA, and the exposure is more sufficient. If medial support is selected, the medial approach will stimulate the soft tissue less than the deltopectoral approach.

There are still several deficiencies in this research. First, the influence of age, gender, occupation and races affected the anatomical structure is not considered. Other specifications were also ignored for the scope of this study, including how height, weight correlate to the distances measured. Second, the average area of exposure of deltoid-splitting, deltopectoral approaches were 1404.39 mm^2^, 1325.41mm^2^, respectively [39]. This article replaced the extent of exposure by the distance between ACHA and PCHA. The precise extent of exposure from the medial approach remains to be studied. Third, results are limited by the number of specimens and measurement errors. Accurate assessment of risk requires more clinical validation.

## Conclusion

The medial approach opens a new perspective in the optimal management of complex fractures of proximal humerus. Anatomical research proves that the medial approach is feasible. The interval between ACHA and PCHA is suitable for placement. Anatomical pattern and indication have been discussed, and we hypothesized that ACHA has been destroyed in complex PHFs. With further studies on the anatomy and mechanism of injury, the development of more clinical cases will be an important work of our institution in the future.

## Data Availability

The datasets used and analyzed during the current study are available from the corresponding author on reasonable request.

## Abbreviations

ACHA: Anterior circumflex humeral artery
PCHA: Posterior circumflex humeral artery
PHF: Proximal humeral fracture
DBA: Deep brachial artery
SSA: Subscapular artery
ORIF: Open reduction and internal fixation
IMN: Intra-medullary nailing
RSA: Reverse shoulder arthroplasty
AVN: Avascular necrosis

## References

[1] Court-Brown CM, Caesar B (2006). Epidemiology of adult fractures: a review. Injury.

[2] Passaretti D (2017). Epidemiology of proximal humeral fractures: a detailed survey of 711 patients in a metropolitan area. J Shoulder Elbow Surg.

[3] Iglesias-Rodriguez S (2021). Epidemiology of proximal humerus fractures. J Orthop Surg Res.

[4] Roux A (2012). Epidemiology of proximal humerus fractures managed in a trauma center. Orthop Traumatol Surg Res.

[5] Palvanen M (2006). Update in the epidemiology of proximal humeral fractures. Clin Orthop Relat Res.

[6] Bahrs C (2014). Trends in epidemiology and patho-anatomical pattern of proximal humeral fractures. Int Orthop.

[7] Klug A (2019). Trends in surgical management of proximal humeral fractures in adults: a nationwide study of records in Germany from 2007 to 2016. Arch Orthop Trauma Surg.

[8] Zhu Y (2011). Locking intramedullary nails and locking plates in the treatment of two-part proximal humeral surgical neck fractures: a prospective randomized trial with a minimum of three years of follow-up. J Bone Joint Surg Am.

[9] Wong J, Newman JM, Gruson KI (2016). Outcomes of intramedullary nailing for acute proximal humerus fractures: a systematic review. J Orthop Traumatol.

[10] Congia S (2020). Is antegrade nailing a proper option in 2- and 3-part proximal humeral fractures?. Musculoskelet Surg.

[11] Lanting B (2008). Proximal humeral fractures: a systematic review of treatment modalities. J Shoulder Elbow Surg.

[12] Gupta AK (2015). Surgical management of complex proximal humerus fractures-a systematic review of 92 studies including 4500 patients. J Orthop Trauma.

[13] Ring D (2007). Current concepts in plate and screw fixation of osteoporotic proximal humerus fractures. Injury.

[14] Gerber C, Werner CM, Vienne P (2004). Internal fixation of complex fractures of the proximal humerus. J Bone Joint Surg Br.

[15] Helmy N, Hintermann B (2006). New trends in the treatment of proximal humerus fractures. Clin Orthop Relat Res.

[16] Micic ID (2009). Analysis of early failure of the locking compression plate in osteoporotic proximal humerus fractures. J Orthop Sci.

[17] Barlow JD (2020). Locking plate fixation of proximal humerus fractures in patients older than 60 years continues to be associated with a high complication rate. J Shoulder Elbow Surg.

[18] Sproul RC (2011). A systematic review of locking plate fixation of proximal humerus fractures. Injury.

[19] Jung SW (2015). Factors that influence reduction loss in proximal humerus fracture surgery. J Orthop Trauma.

[20] Gardner MJ (2007). The importance of medial support in locked plating of proximal humerus fractures. J Orthop Trauma.

[21] Zhang X (2019). Inferomedial cortical bone contact and fixation with calcar screws on the dynamic and static mechanical stability of proximal humerus fractures. J Orthop Surg Res.

[22] Park SG, Ko YJ (2019). Medial buttress plating for humerus fractures with unstable medial column. J Orthop Trauma.

[23] He Y (2017). Biomechanical evaluation of a novel dualplate fixation method for proximal humeral fractures without medial support. J Orthop Surg Res.

[24] Wang F (2021). A novel surgical approach and technique and short-term clinical efficacy for the treatment of proximal humerus fractures with the combined use of medial anatomical locking plate fixation and minimally invasive lateral locking plate fixation. J Orthop Surg Res.

[25] Chen YX (2014). Anatomical study of simple landmarks for guiding the quick access to humeral circumflex arteries. BMC Surg.

[26] Yongliang Y (2020). Intraoperative ultrasonography assistance for minimally invasive repair of the acute Achilles tendon rupture. J Orthop Surg Res.

[27] Olinger A, Benninger B (2010). Branching patterns of the lateral thoracic, subscapular, and posterior circumflex humeral arteries and their relationship to the posterior cord of the brachial plexus. Clin Anat.

[28] Brooks CH, Revell WJ, Heatley FW (1993). Vascularity of the humeral head after proximal humeral fractures. An anatomical cadaver study. J Bone Joint Surg Br.

[29] Duparc F, Muller JM, Freger P (2001). Arterial blood supply of the proximal humeral epiphysis. Surg Radiol Anat.

[30] Gerber C, Schneeberger AG, Vinh TS (1990). The arterial vascularization of the humeral head. An anatomical study. J Bone Joint Surg Am.

[31] Hettrich CM (2010). Quantitative assessment of the vascularity of the proximal part of the humerus. J Bone Jt Surg Am.

[32] Keough N (2019). An anatomical investigation into the blood supply of the proximal humerus: surgical considerations for rotator cuff repair. JSES Open Access.

[33] Hagiwara Y (2015). Blood flow changes of the anterior humeral circumflex artery decrease with the scapula in internal rotation. Knee Surg Sports Traumatol Arthrosc.

[34] Sergent A (2020). Quantitative localization of the entry point of the lateral ascending branch of the anterior circumflex humeral artery: a high definition CT-scan radiological study. Surg Radiol Anat.

[35] Gates S (2020). Surgically relevant anatomy of the axillary and radial nerves in relation to the latissimus dorsi tendon in variable shoulder positions: a cadaveric study. Shoulder Elbow.

[36] Kjelstrup T, Sauter AR, Hol PK (2017). The relationship of the musculocutaneous nerve to the brachial plexus evaluated by MRI. J Clin Monit Comput.

[37] Boileau P (2018). Isolated loss of active external rotation: a distinct entity and results of L'Episcopo tendon transfer. J Shoulder Elbow Surg.

[38] Hertel R (2004). Predictors of humeral head ischemia after intracapsular fracture of the proximal humerus. J Shoulder Elbow Surg.

[39] Sirisreetreerux N, Pengrung N, Apivatthakakul T (2021). Proximal humerus exposure with the inverted-L anterolateral deltoid flip approach, anterolateral deltoid splitting approach, and deltopectoral approach: a comparative cadaveric study. Injury.

